# Designing Programs for Eliminating Canine Rabies from Islands: Bali, Indonesia as a Case Study

**DOI:** 10.1371/journal.pntd.0002372

**Published:** 2013-08-22

**Authors:** Sunny E. Townsend, I Putu Sumantra, Pudjiatmoko, Gusti Ngurah Bagus, Eric Brum, Sarah Cleaveland, Sally Crafter, Ayu P. M. Dewi, Dewa Made Ngurah Dharma, Jonathan Dushoff, Janice Girardi, I Ketut Gunata, Elly F. Hiby, Corlevin Kalalo, Darryn L. Knobel, I Wayan Mardiana, Anak Agung Gde Putra, Luuk Schoonman, Helen Scott–Orr, Mike Shand, I Wayan Sukanadi, Pebi Purwo Suseno, Daniel T. Haydon, Katie Hampson

**Affiliations:** 1 Boyd Orr Centre for Population and Ecosystem Health, Institute for Biodiversity, Animal Health and Comparative Medicine, College of Medical, Veterinary and Life Sciences, University of Glasgow, Glasgow, Scotland, United Kingdom; 2 Bali Province Livestock Services, Jalan Asoka no. 14, Kreneng, Denpasar, Bali, Indonesia; 3 Directorate General of Livestock and Animal Health Services, Ministry of Agriculture of the Republic of Indonesia, Jalan Harsono, Jakarta, Indonesia; 4 Bali Animal Welfare Association, Ubud, Bali, Indonesia; 5 Food and Agriculture Organization (FAO), Emergency Centre for Transboundary Animal Diseases (ECTAD), Jakarta, Indonesia; 6 Disease Investigation Center Denpasar, Denpasar, Bali, Indonesia; 7 Department of Biology, McMaster University, Hamilton, Ontario, Canada; 8 Badung District Livestock Services, Jalan Raya Sempidi, Mangupura, Badung, Bali, Indonesia; 9 World Society for the Protection of Animals, London, United Kingdom; 10 Department of Veterinary Tropical Diseases, Faculty of Veterinary Science, University of Pretoria, Onderstepoort, South Africa; 11 Faculty of Veterinary Science, University of Sydney, Camden, New South Wales, Australia; 12 School of Geographical and Earth Sciences, University of Glasgow, Glasgow, Scotland, United Kingdom; The Global Alliance for Rabies Control, United States of America

## Abstract

**Background:**

Canine rabies is one of the most important and feared zoonotic diseases in the world. In some regions rabies elimination is being successfully coordinated, whereas in others rabies is endemic and continues to spread to uninfected areas. As epidemics emerge, both accepted and contentious control methods are used, as questions remain over the most effective strategy to eliminate rabies. The Indonesian island of Bali was rabies-free until 2008 when an epidemic in domestic dogs began, resulting in the deaths of over 100 people. Here we analyze data from the epidemic and compare the effectiveness of control methods at eliminating rabies.

**Methodology/Principal Findings:**

Using data from Bali, we estimated the basic reproductive number, *R*_0_, of rabies in dogs, to be ∼1·2, almost identical to that obtained in ten–fold less dense dog populations and suggesting rabies will not be effectively controlled by reducing dog density. We then developed a model to compare options for mass dog vaccination. Comprehensive high coverage was the single most important factor for achieving elimination, with omission of even small areas (<0.5% of the dog population) jeopardizing success. Parameterizing the model with data from the 2010 and 2011 vaccination campaigns, we show that a comprehensive high coverage campaign in 2012 would likely result in elimination, saving ∼550 human lives and ∼$15 million in prophylaxis costs over the next ten years.

**Conclusions/Significance:**

The elimination of rabies from Bali will not be achieved through achievable reductions in dog density. To ensure elimination, concerted high coverage, repeated, mass dog vaccination campaigns are necessary and the cooperation of all regions of the island is critical. Momentum is building towards development of a strategy for the global elimination of canine rabies, and this study offers valuable new insights about the dynamics and control of this disease, with immediate practical relevance.

## Author Summary

Canine rabies continues to cause tens of thousands of horrific deaths worldwide, primarily in Asia and Africa. Momentum is building towards development of a global elimination strategy for canine rabies, but questions remain over how best to eliminate rabies epidemics. This paper uses data generated from the recent high-profile rabies outbreak in Bali, Indonesia to evaluate different control options. We find that, despite high dog densities, the spread of rabies on the island was remarkably similar to canine rabies spread elsewhere, suggesting that the practice of dog culling is an ineffective control strategy. We then simulate rabies transmission and spread across the island and compare the effectiveness of mass dog vaccination strategies in terms of how many lives are saved and how long it will take for elimination to be achieved. We find that the effectiveness of campaigns is not improved by being more reactive or synchronized but depends almost entirely upon reaching sufficient coverage (70%) across the population in successive campaigns. Even small ‘gaps’ in vaccination coverage can significantly impede the prospects of elimination. The outputs of this study provide the kind of evidence needed by rabies program coordinators to help design effective national control programmes, and to build the evidence-base to drive forward the development and implementation of effective global rabies policy.

## Introduction

Rabies transmitted by domestic dogs is a re–emerging public health problem in Asia. In recent years incidence has increased dramatically in China [1], [2]; multiple incursions have been reported from Bhutan [3], [4]; and the disease has spread across several previously rabies–free islands in Indonesia (Flores 1997 [5], Maluku 2003, North Maluku 2005, West Kalimantan 2005, Nias 2009 [6]), including the popular tourist destination of Bali [7].

**Figure 1:**
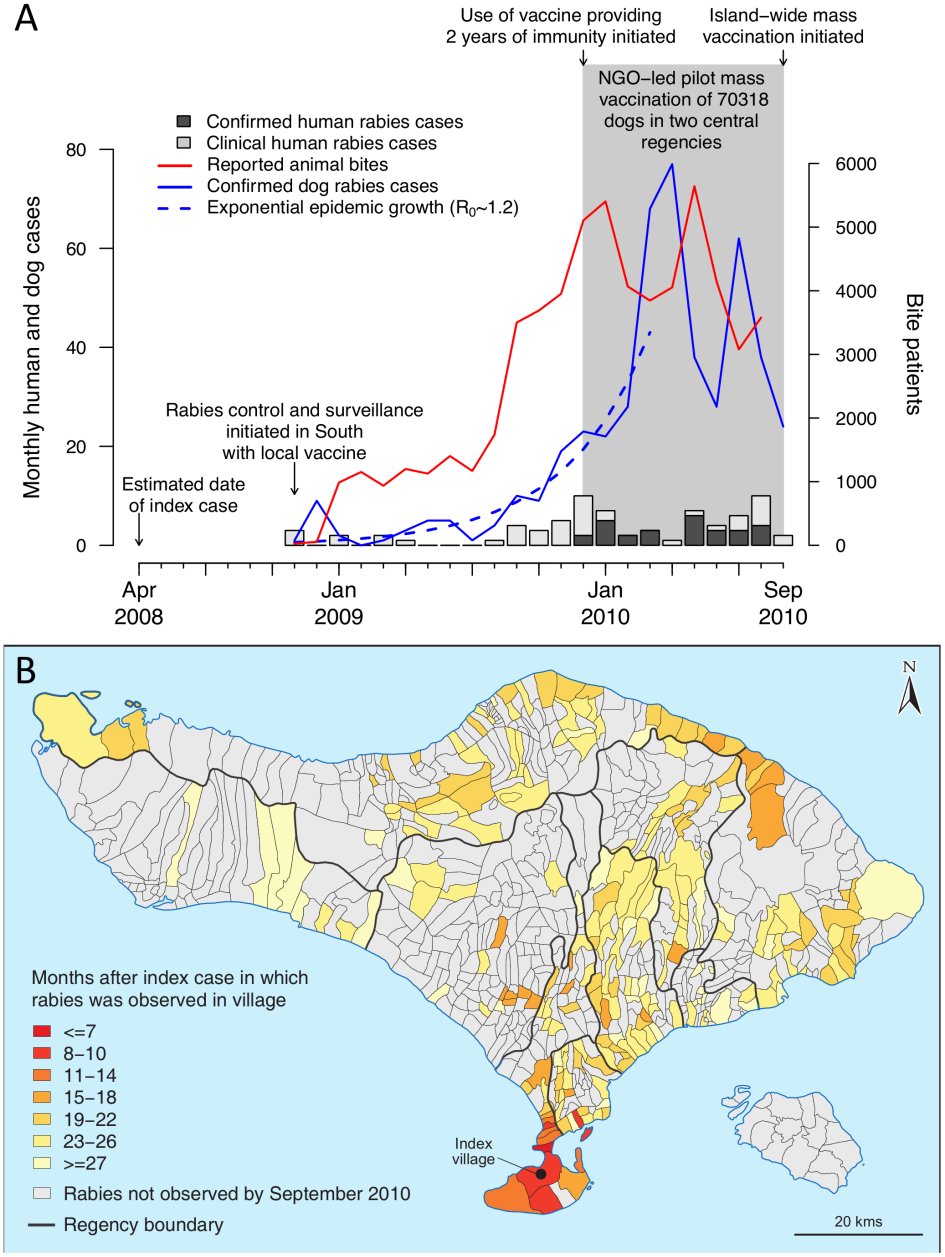
Rabies incidence and spread in Bali prior to island-wide mass vaccination. (A) Cases in humans and dogs and corresponding control efforts. (B) The month that rabies was first confirmed in each village. The black dot marks the village where the index case occurred. Regencies are outlined in black.

The island province of Bali was historically rabies–free until late 2008, when several local people died in the southernmost peninsula showing signs of the disease. An incursion is thought to have occurred approximately seven months earlier, when a fisherman landed on the peninsula with a dog that was incubating the virus [8]. Initial control efforts by the Balinese government attempted to contain the outbreak to the two administrative districts (Regencies) within the peninsula. However, in August 2009 a human case was diagnosed beyond the outbreak locality, and by July 2010 cases had been confirmed in all nine Regencies of Bali and 62 people had died (Fig. 1). As is common with an unexpected incursion: the island lacked surveillance, medical staff trained in rabies diagnosis, and contingency planning. The ensuing epidemic generated local and international pressure to eradicate rabies and led to plans for island–wide mass vaccination of the dog population (ProMED-mail archive number 20100806.2673).

The government's main concern for the effectiveness of any proposed rabies control programme on Bali was the high density of domestic dogs. Dogs are an important part of Balinese culture; the majority of households own at least one dog [9], though most are unconfined and not easy to restrain for parenteral vaccination. However, a pilot vaccination campaign that used trained dog–catchers equipped with nets showed that more than 80% of dogs could be vaccinated [10], with a team of six vaccinating around 100 dogs per day (Fig. 1A). From initial estimates of the human∶dog ratio (8∶1) the Bali dog population was extrapolated to be 400,000, with densities exceeding 250 km^−2^ in urban areas [11]. The basic reproductive number, *R*_0_, measures the average number of secondary cases arising from a primary infected individual in an otherwise fully susceptible population, and determines the critical level of vaccination coverage needed to protect the population (‘herd immunity’) and bring a disease under control [12]. For directly transmitted diseases such as rabies, *R*_0_ is often assumed to depend on population density [12], implying that such high–density dog populations could limit the success of mass vaccination. Estimating *R*_0_ for rabies on Bali was therefore a priority for determining whether vaccination would be a feasible control strategy and for setting coverage targets. The relationship between dog rabies incidence and human rabies deaths was a further important consideration for estimating public health impacts of proposed strategies.

Considerable successes have been achieved in the control of rabies in many parts of the world through the mass vaccination of domestic dogs [13], [14], [15], [16] and mounting evidence demonstrates that regional elimination of canine rabies is possible through sustained annual campaigns that attain 70% coverage [17], [18]. However, there are no operational guidelines on how to roll out dog vaccination campaigns strategically in the face of an emerging epidemic. We developed a model to capture the inherent variation in epidemic trajectories, particularly as eradication is approached, to guide strategic choices in planning Bali's first island–wide mass vaccination campaign. We fitted dog rabies incidence to human deaths in order to link the model output to potential human deaths averted. The model addressed concerns over the extremely dense population of dogs and presumed high levels of dog population turnover. We used the model to investigate whether vaccination campaigns that reach 70% of dogs on Bali could provide herd immunity, and how many campaigns would be needed to achieve eradication. We investigated how campaign effectiveness might be affected by use of locally–produced (potentially more affordable and sustainable) vaccines versus longer–acting, imported vaccines, by the speed of delivery and strategic rolling out of the programme across Bali and by the interval between campaigns. Then we examined how robust campaign performance would be to human–mediated movement of dogs around Bali and heterogeneities in coverage arising from political, logistical and operational constraints. Finally we explored the impacts of the vaccination campaigns that have since been implemented on Bali and their prospects for achieving eradication, and provide advice for how these prospects may be enhanced.

## Methods

### The model

**Figure 2:**
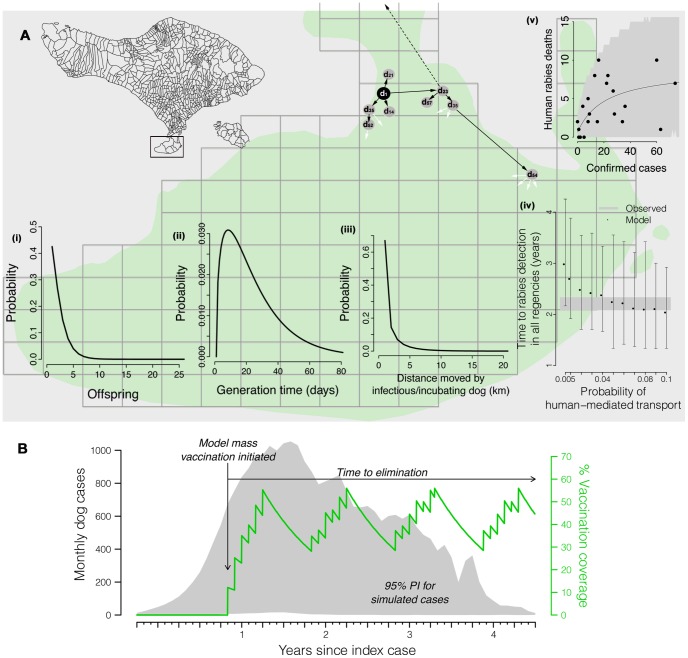
Model description. (A) Secondary cases are drawn from the (i) offspring distribution, and become infectious at a date drawn from the (ii) generation interval distribution: here four secondary cases are generated by the index case (black dot) which become infectious on day 14, 21, 23, and 35. The occurrence of secondary cases depends on vaccination coverage in the grid cell at the time of transmission. (iii) With probability 1–*p* each offspring occurs at a location generated from the local dispersal kernel (solid black arrows). (iv) With probability *p*, each offspring occurs on any randomly chosen grid cell (broken black arrow). It took 2.2 years for rabies to be detected in all nine Regencies (grey band), consistent with *p* = 0.05–0.09 (black dots are medians with 95% PIs from 100 simulations). See Table 1 for parameterization of distributions. (v) Human rabies deaths versus confirmed dog rabies cases, showing the best-fit relationship (black line, see Results for equation) and 95% confidence intervals (grey area). (B) 95% PI envelope of simulated cases (grey area) with annual campaigns of the ‘random’ mass vaccination strategy (green line, Table 2), which is rolled out when cumulative cases reach 7,000 and from which point the time to eradication is measured.

Figure 2 provides a visual summary of the model of dog-dog transmission and spread across Bali, as well as the functional form used to predict human rabies cases. We assumed that each infectious dog case causes κ secondary dog cases (‘offspring’), drawn from a negative binomial distribution (κ∼negative binomial(*R*_0_, *k*), Table 1, Fig. 2Ai), with *R*_0_ as its mean [20], [21]. Each secondary case was assigned a generation interval selected from a gamma distribution [18] (Table 1, Fig. 2Aii) representing an incubation period plus a period of infection prior to transmission, to determine when new infections were generated. Using an explicit spatial representation of Bali based on 1 km^2^ grid cells (Fig. 2A), we probabilistically allocated the location of each secondary case. To capture human–mediated transport of dogs across the island, exposed offspring were assigned to a randomly chosen grid cell with probability *p* so that infected dogs could potentially travel much further distances than a rabid dog is capable of running. To capture the local movement of rabid dogs, secondary cases were displaced from their direct epidemiological predecessors according to a gamma–distributed dispersal kernel [18] (Table 1, Fig. 2Aiii), with probability 1–*p*.

**Table 1:** Parameters values and distributions used to model rabid dog movement and transmission processes.

Process	Distribution	Parameter	Value	Source
Generation interval	Gamma	Mean Variance	24 days380 days	Based on best fit parameters (shape = 1.46 and scale = 16.1) [18]
Reproductive number *R*_0_	Negative binomial	Mean Dispersion parameter *k*	1·201·33	See Methods [18]
Local movement spatial kernel	Gamma	Mean Variance	0·88 km3.58 km	Based on best fit parameters (shape = 0.215 and rate = 0.245) [18]
Probability of human–mediated dog transport		*p*	0·05	Figure 2Aiv
Vaccination coverage			70%	Assumption (see *Sensitivity to parameters* & Fig. 3B)
Annual dog population turnover			50%	Assumption (see *Sensitivity to parameters* & Fig. 3C)
Duration of immunity			2 years	Assumption (see *Sensitivity to parameters* & Fig. 3D)
Probability of confirming a dog case			0.07	[7]

We estimated the initial epidemic growth rate *λ* from the monthly time series of confirmed dog rabies cases (Fig. 1A) using a generalized linear model with negative binomial errors [18]. We converted the inferred initial epidemic growth rate to an estimate of *R*_0_ using the probability distribution function of the generation interval (*G_t_*) for rabies based on data from natural infections [18], according to Wallinga & Lipstich [22]:
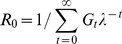



The *R*_0_ estimate for Bali was used as the mean of the offspring distribution in the model (Fig. 2Ai).

To estimate the relationship between confirmed dog rabies cases and human deaths, we fitted several functions using maximum likelihood and used AIC to select the best fitting model (Fig. 2Av).

The probability of human-mediated transport of dogs across the island, *p*, was inferred by incrementally increasing the proportion of rabid and incubating dogs that were moved randomly across the island until the modelled speed of spread matched the observed spread of the epidemic (Fig. 2Aiv, assuming a case detection probability of 0.07 [6]). Other parameters used in the model (Table 1, Fig. 2) were derived from epidemiological data on naturally infected rabid dogs in Tanzania [18].

We modeled vaccination coverage (the proportion of dogs vaccinated, *V*) in each cell as waning exponentially from the coverage achieved at the time of vaccination, at a rate (*Δt* = one day) determined by dog population turnover (*b* = birth rate and death rate, assuming constant population size) and the duration of the vaccine–induced immunity (*τ*, where *v* = 1/*τ*):
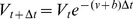
Parameter estimates are provided in Table 1. We made the conservative assumption that coverage did not accumulate over multiple vaccination campaigns (see Supporting Information for more details). Dog vaccination is represented in the model by reducing the number of secondary cases per primary infection in direct proportion to vaccination coverage at the time of transmission. In effect, each potential secondary case becomes infectious with probability 1−*V_t_*, so in a vaccinated population the number of secondary cases attributed to each case is κ_v_∼binomial(κ,*V_t_*).

The branching process formulation does not account for any effects of depletion of the susceptible population as disease incidence increases. However, since detected incidence on Bali did not exceed 0.2% per annum, depletion of the susceptible population is assumed to play a negligible part. Likewise, we did not include the effects of rabies incidence on the proportion of dogs vaccinated.

The island–wide mass vaccinations on Bali began in October 2010 by which time 477 cases of rabies had been confirmed in dogs. We suspect that samples were retrieved from less than 10% of rabies cases (based on [6], [23] and previous experience during intensive contact tracing studies in northern Tanzania that suggest samples are recovered from around 5–10% of identified cases), therefore we commence vaccination in the model after 7,000 cases had occurred in model realizations. We assume the vaccinations failed to eradicate rabies if 40,000 cases were reached. The expected behavior of the epidemic under alternative scenarios was estimated using two measures: 1) the probability of eradication of rabies from Bali, and 2) the time to eradication from the onset of vaccination. For each scenario we ran 1,000 realizations of the model.

Statistical analyses were carried out in R (version 2.14.2, R Core Team 2012) and the model was built in MATLAB (version 7 release 14, The MathWorks Inc.). Codes are available upon request to the corresponding author.

### Sensitivity to parameters

We explored the sensitivity of performance measures to variation in *R*_0_ (between 1 and 2 based on estimates of *R*_0_ from rabies outbreaks around the world [18]), vaccination coverage; domestic dog population turnover (assuming constant population size and birth/death rates varying from 0.1 to 2.3 year^−1^ spanning a range of population replacement from 10% to 90% per year); duration of vaccine–induced immunity; and variation in long-distance dog movement, to investigate the potential impact of restrictions on human–mediated transport of exposed or infected dogs.

### Vaccination strategies

The island grid was aggregated into 24 rectangular blocks of similar size (mean 277 km^2^, range 49–500 km^2^) to evaluate strategies. We analyzed repeat campaigns (1, 2 and 3 campaigns) under a range of coverage levels (40%, 60% or 80%) and inter–campaign intervals (0, 6 or 12 months). We considered one synchronous campaign vaccinating all 24 blocks in the same month (A in Table 2), four proactive strategies each of six–month duration vaccinating four blocks each month in different sequences (random, rotate, source and furthest, B–E in Table 2) and two reactive strategies of six–month duration (F–G in Table 2). To examine the impact of heterogeneity in vaccination coverage we compared the effect of leaving unvaccinated areas distributed across the island in two ways: either randomly distributed unvaccinated 1 km^2^ grid cells, or equivalently–sized contiguous blocks of unvaccinated grid cells. Videos of model simulations of a sample of the scenarios we considered are available as Supporting Information.

**Table 2:** Modelled vaccination strategies.

	Vaccination strategy label used in Fig. 5B	Description	Campaign duration
A	Sync	Synchronous vaccination of the island in one month	1 month
B	Random	Random ordering of blocks	6 months
C	Rotate (Video S1)	Start in the center of the island and rotate anticlockwise, ending in the southern peninsula	6 months
D	Source	Start in the southern peninsula where the index case occurred and vaccinate contiguously northward	6 months
E	Furthest	Start on the West coast (furthest point from southern peninsula) and vaccinate contiguously eastward	6 months
F	Reactive	Prioritize blocks with the highest number of cases in preceding month, vaccinate up to 4 blocks in each month	6 months
G	React w/o repeat (Video S2)	As for the Reactive strategy except does not permit revaccination of blocks within the same 6 month campaign	6 months
H	Actual (Fig. 6, Video S5)	Vaccination that took place in Bali between November 2008 and December 2011	-

All campaigns were annual unless specified in the analyses.

### The vaccination campaigns of Bali

To estimate vaccination coverages achieved in Bali, data on vaccination dates, numbers of dogs vaccinated and post-vaccination surveys (counts of dogs with or without collars signifying vaccination) were compiled at the banjar (sub-village) level, where possible. Where data were only available at courser resolution, numbers of dogs vaccinated were split between corresponding villages and banjars. Dog population size was calculated from post-vaccination surveys in banjars as: dogs vaccinated/(collared dog count/total dogs counted). If surveys were not available, dog populations were estimated from the human∶dog ratio for the village, district or regency as available. To obtain vaccination coverages by 1 km^2^ grid cell, banjar centroids were assigned randomly within their village polygon, and coverage averaged from banjar centroids within the grid cell or, if empty, assigned from the nearest banjar centroid. We assumed lakes, reservoirs, forested areas and mountain peaks were not inhabited by dogs. Coverage was assumed to wane as described above, and epidemic trajectories were simulated across the resulting dynamic coverage landscape.

## Results

During the course of the Bali outbreak, suspect cases of dogs with rabies that had either bitten people, other animals, or had shown clinical signs of disease were reported to local veterinary laboratories. Where possible such animals were captured and quarantined for observation, though many were culled. Brain samples from animals that had died, been culled or euthanized in quarantine, were tested using the direct fluorescent antibody test to confirm the presence of rabies. These data were collated by month to generate a time series of confirmed dog rabies cases (Fig. 1A). The basic reproductive number (*R*_0_) for rabies on Bali was estimated to be 1.2 (95% percentile interval 1.0–1.3) based on the epidemic trajectory until the peak in April 2010 (Fig. 1A, see the Supporting Information for definition of a percentile interval (PI)). Regency–specific *R*_0_ estimates varied between 1.0 and 1.5.

We developed a model to capture the variation in biting behavior and movement of infectious dogs. The model was a spatially explicit, stochastic simulation of rabies spread based on a simple branching density–independent process (Fig. 2, videos of simulations are available as Supporting Information). Based on confirmed cases and the estimated date of the index case, it took 26 months, April 2008–June 2010, for rabies to be detected in all nine Regencies of Bali (Fig. 1B). We tuned the probability *p* of longer distance human–mediated transport of infectious/incubating animals in the model until the modelled and observed speed of epidemic spread matched (Fig. 2Aiv) estimating *p* to lie between 0.05 and 0.09 during this initial phase of the epidemic (Fig. 2Aiv). The best fit relationship between monthly confirmed dog rabies cases (*D*) and monthly human deaths (*H*) was a saturating functional response (Fig. 2Av) with negative binomial errors (*k* = 3.697):
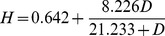



### Sensitivity to parameters

**Figure 3:**
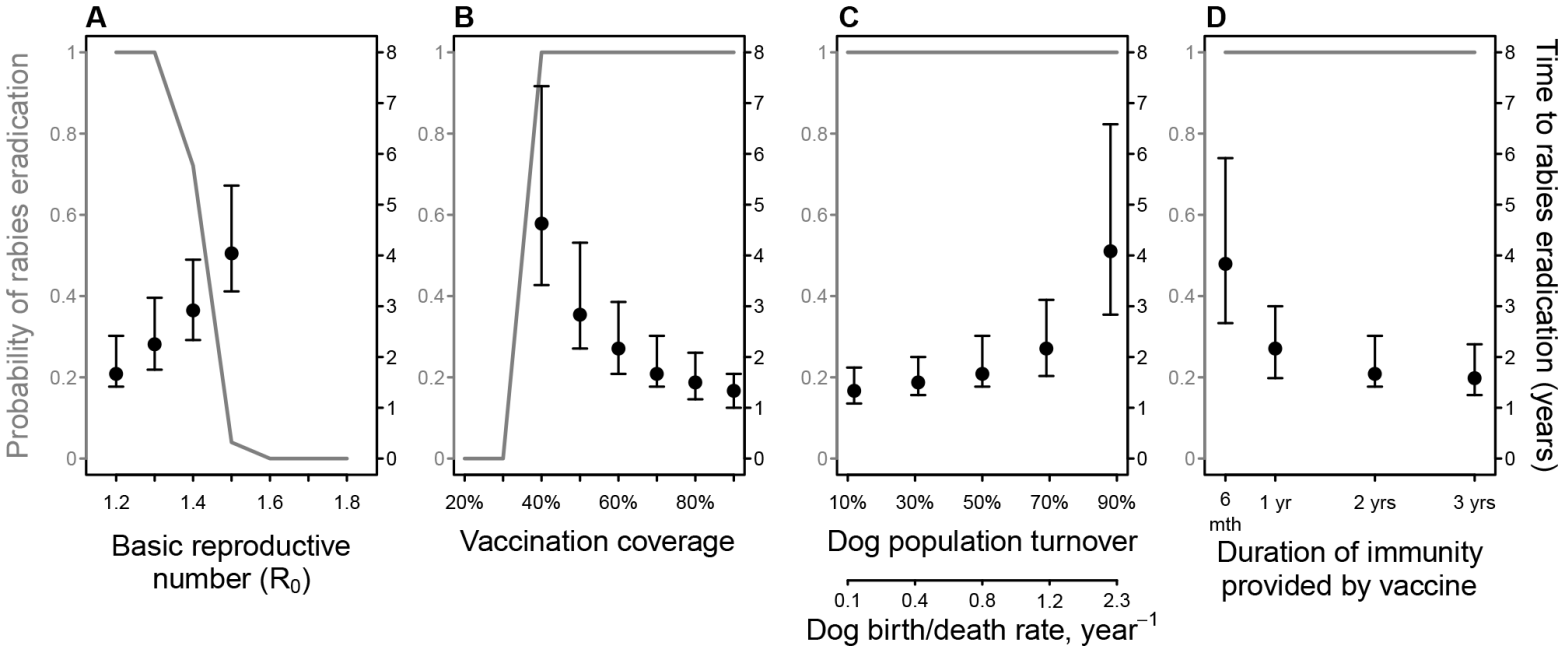
Key epidemiological and operational variables determining the success of rabies vaccination programmes in terms of the predicted probability of eradication (grey y–axis and line) and time to eradication (black y–axis, medians and 95% PI), showing sensitivity to: (A) the basic reproductive number, *R*_0_, (B) vaccination coverage (achieved at the time and location of the campaign (see Fig. 4)), (C) annual dog population turnover, with conversion into birth/death rate assuming constant population size (birth rates equal to death rates), and (D) duration of immunity provided by vaccine. Based on 1000 simulations generated using parameters in Table 1 (unless specified) and annual campaigns of the ‘random’ mass vaccination strategy (Table 2).

**Figure 4:**
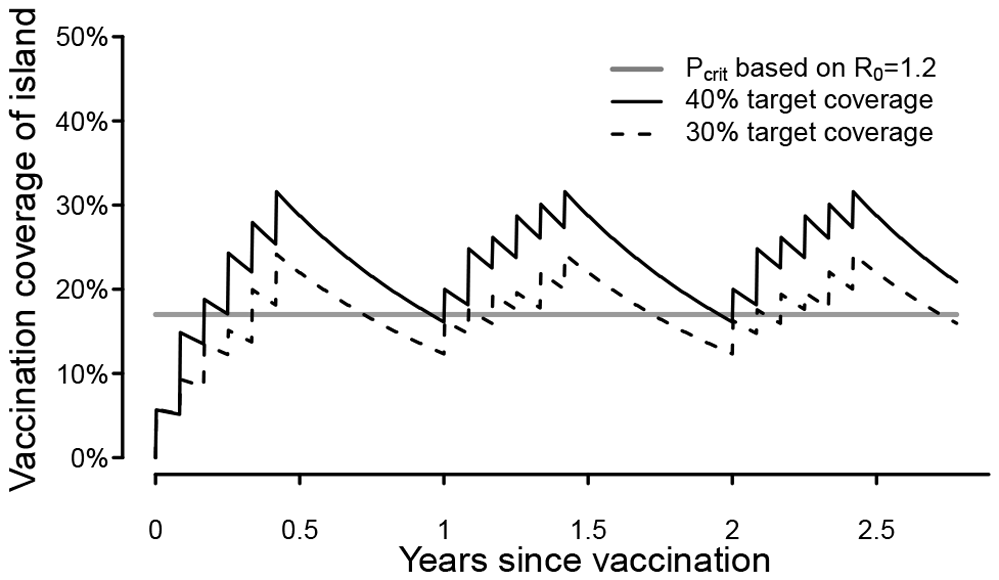
Trajectories of vaccination coverage achieved at the island-wide level during modeled vaccination campaigns and in relation to levels of coverage required for herd immunity. Three types of coverage are referred to in the text: target coverage achieved in the subset of the population at the time and location of a local campaign (i.e. within a block); island-wide vaccination coverage (y-axis); and critical vaccination coverage (*P_crit_*) which is required for herd immunity and is determined by *R*_0_, the basic reproductive number of rabies in Bali, *P_crit_* = 1-(1/*R*_0_). *R*_0_ estimated for Bali is 1·2, which corresponds to a *P_crit_* of 17% (grey solid line). A 40% coverage campaign resulted in a trajectory that stayed above 17% (black solid line) and the probability of eradication was 1 (Fig. 3B), whereas 30% coverage resulted in a trajectory that dipped below 17% (black dashed line) and the probability of eradication was less than 1 (Fig. 3B). Annual campaigns were modeled, using parameters in Table 1 and the ‘random’ six-month strategy (Table 2). Blocks are assumed to be vaccinated at the end of the month hence coverage increments jaggedly. Coverage declines between vaccinations due to waning of immunity and dog population turnover.

We observed an exponential relationship between modeled *R*_0_ and the median time to rabies eradication (Fig. 3A). Above a threshold value (*R*_0_ between 1.3 and 1.4), the probability of eradication fell to below one even for annual campaigns that achieved 70% coverage (Fig. 3A). When *R*_0_ was equal to 1.2, vaccination programmes with annual campaigns eventually eradicated rabies if coverage targets of at least 40% were met (Fig. 3B, Fig. 4). If campaigns achieved the WHO–recommended target of 70% coverage, the probability of eradication was largely insensitive to population turnover and duration of vaccine–induced immunity. Only at the highest turnover rates (>70%) and shortest vaccination immunity durations (<1 year) was the time to eradication substantially prolonged (Fig. 3C&D).

### Vaccination strategies

**Figure 5:**
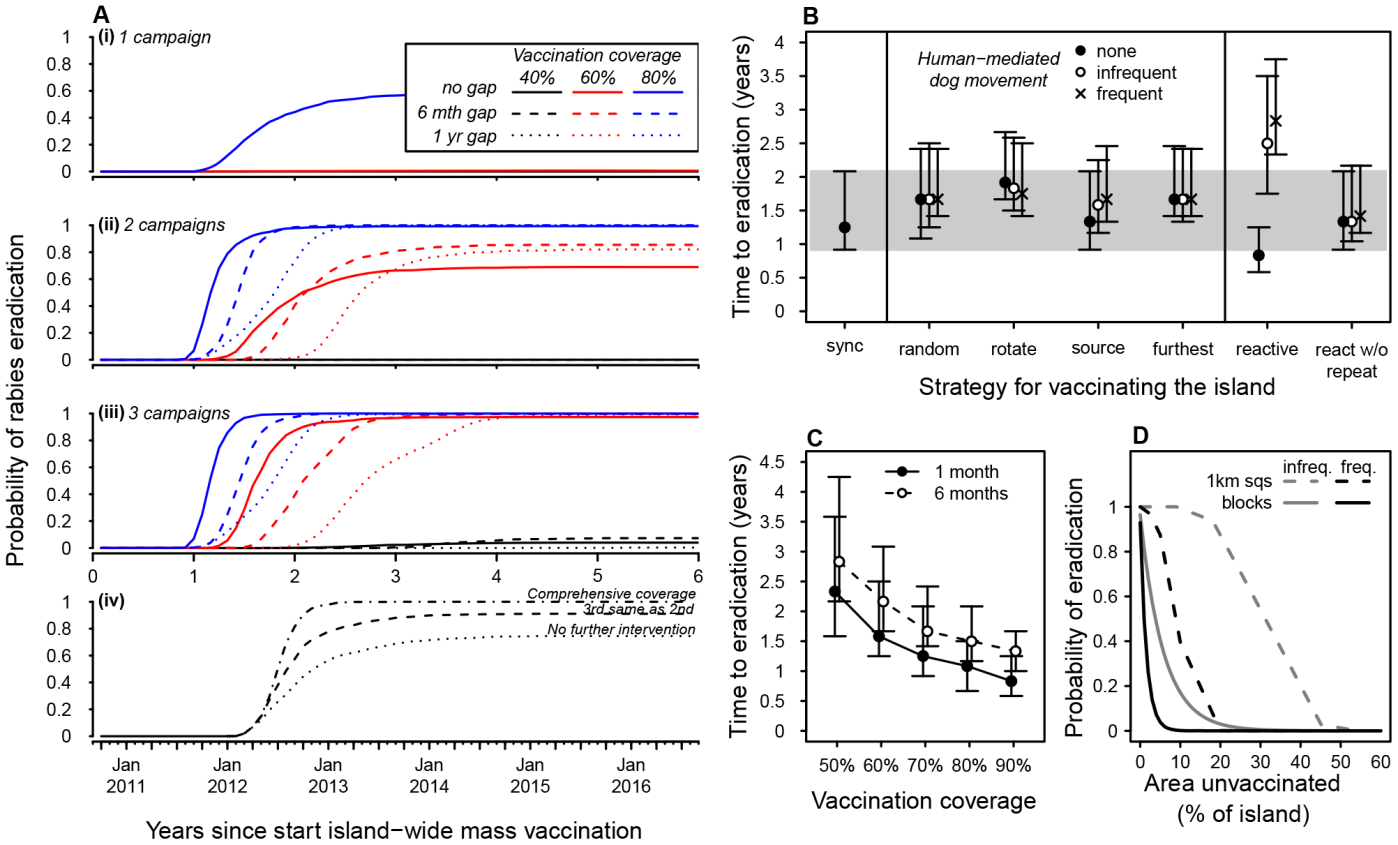
Vaccination strategies. The probability of eradication following: (Ai) 1; (Aii) 2; (Aiii) 3 campaigns under a range of coverages (40, 60, 80%) and inter–campaign intervals (0, 6, 12 months); (Aiv) vaccination as implemented on Bali, and projected from January 2012 when rabies was still circulating. The time to eradication (medians with 95% PI) for a range of: (B) frequencies of human–mediated transport of dogs (*p* = 0, 0.02 or 0.05) and campaign strategies (Table 2). 95% PI of the one-month ‘sync’ strategy is highlighted (grey band) for comparison with the six–month strategies; (C) coverages when campaigns last 1 month or 6 months. (D) The probability of eradication with % island area left unvaccinated, made up of either randomly chosen 1 km squares (solid lines) or randomly chosen blocks, and when human-mediated movement of dogs was either infrequent (*p* = 0.02, grey) or frequent (*p* = 0.05, black).

The number of consecutive island–wide annual campaigns and coverage achieved strongly influenced the probability of eradicating rabies (Fig. 5A). A single high coverage (80%) campaign did not guarantee eradication, but had a reasonable probability of success (∼0.6), whereas a single 40% or 60% coverage campaign had no prospect of achieving eradication (Fig. 5Ai). Subsequent campaigns greatly increased eradication prospects: two campaigns of 80% coverage or three campaigns of 60% coverage eradicated rabies in more than 90% of model runs, but three consecutive low coverage (40%) campaigns still had a very low prospect of achieving eradication (Fig. 5Aii & iii). Six consecutive low coverage campaigns increased the likelihood of eradication to ∼90% (Fig. 3B).

Thus, a roughly equivalent reasonable chance of eradication (∼90%) can be achieved with a two high coverage (80%), three annual moderate coverage (60%) or six annual low coverage (40%) campaigns. Increasing campaign frequency did not greatly affect the probability of eradication, but annual campaigns of six–month duration with six–month inter–campaign intervals could be slightly more effective than back–to–back campaigns (Fig. 5Aii & iii).

Based on the pilot vaccinations (Fig. 1A), it was estimated that the methods used could be feasibly scaled–up to cover the entire island within a six–month period, but more intensive vaccinations (1–month synchronized) might compromise coverage because of insufficient availability of trained teams. Completing campaigns in six months rather than one month (‘sync’) delayed eradication by a few months, but these delays could be compensated for by a small increase in coverage (Fig. 5C). Therefore on the basis of six-month long campaigns, we compared strategies for how to vaccinate the island, based upon different patterns of rollout under consideration at the time of planning the first campaign (Table 2 B–E). Time to eradication under different strategies varied depending on the spatial evenness of cases and thus was sensitive to potentially long distance, human–mediated transport of dogs (Fig. 5B). When human-mediated dog movement was restricted or at low frequency (*p* = 0 and 0.02, Fig. 5B) cases were less evenly distributed and the strategy that most rapidly eradicated rabies started vaccinations in the southernmost Regency where the index case occurred (‘source’). In contrast, the strategy that ended in the South (‘rotate’) took longest and the random strategy and the wave–like strategy from West to East (‘furthest’) were intermediate in performance. When human-mediated dog movement was frequent (*p* = 0.05, Fig. 5B) all four strategies performed similarly. We also compared two six-month reactive strategies (Table 2 F–G): the strategy that vaccinated blocks solely based on incidence (‘reactive’), produced the most variation in eradication times (Fig. 5B). This strategy eradicated rabies more rapidly than all others, including the synchronized campaign, when there was no human-mediated dog movement, but took longest when human–mediated movement was frequent (*p* = 0.05). The performance of the reactive strategy that did not return to previously vaccinated blocks within the same campaign (‘react w/o repeat’) was more robust to long distance movement (Fig. 5B).

We looked at the probability of eradicating rabies when there were gaps in coverage and under the scenarios of low and high frequency human-mediated dog movement where dogs could potentially be transported to any point on the island. When human-mediated dog movement was relatively low (*p* = 0.02), and gaps were modelled by excluding randomly distributed 1 km^2^ grid cells during vaccinations, the effect on the probability of eradication was negligible if the total area omitted was less than ∼10% of the island (Fig. 5D) and declined in a roughly linear fashion, reaching 0.9 when ∼20% of the island was not vaccinated (Fig. 5D). In contrast, when the same proportion of unvaccinated cells were left in contiguous blocks, the probability of eradication dropped rapidly, reaching 0.9 when just 0.4% of the island's area was omitted, which equates to just three neighboring villages of Bali's ∼700 villages (Fig. 5D). In both situations, the probability of eradication reaches zero when ∼50% of the island's area is left unvaccinated, but the decline is exponential when unvaccinated grid cells are aggregated (Fig. 5D). More frequent human-mediated dog movement (*p* = 0.05) amplifies the effects of gaps in coverage on the probability of eradication, with a greater chance of rabies reaching and persisting in unvaccinated areas (Fig. 5D).

### The vaccination campaigns of Bali

**Figure 6:**
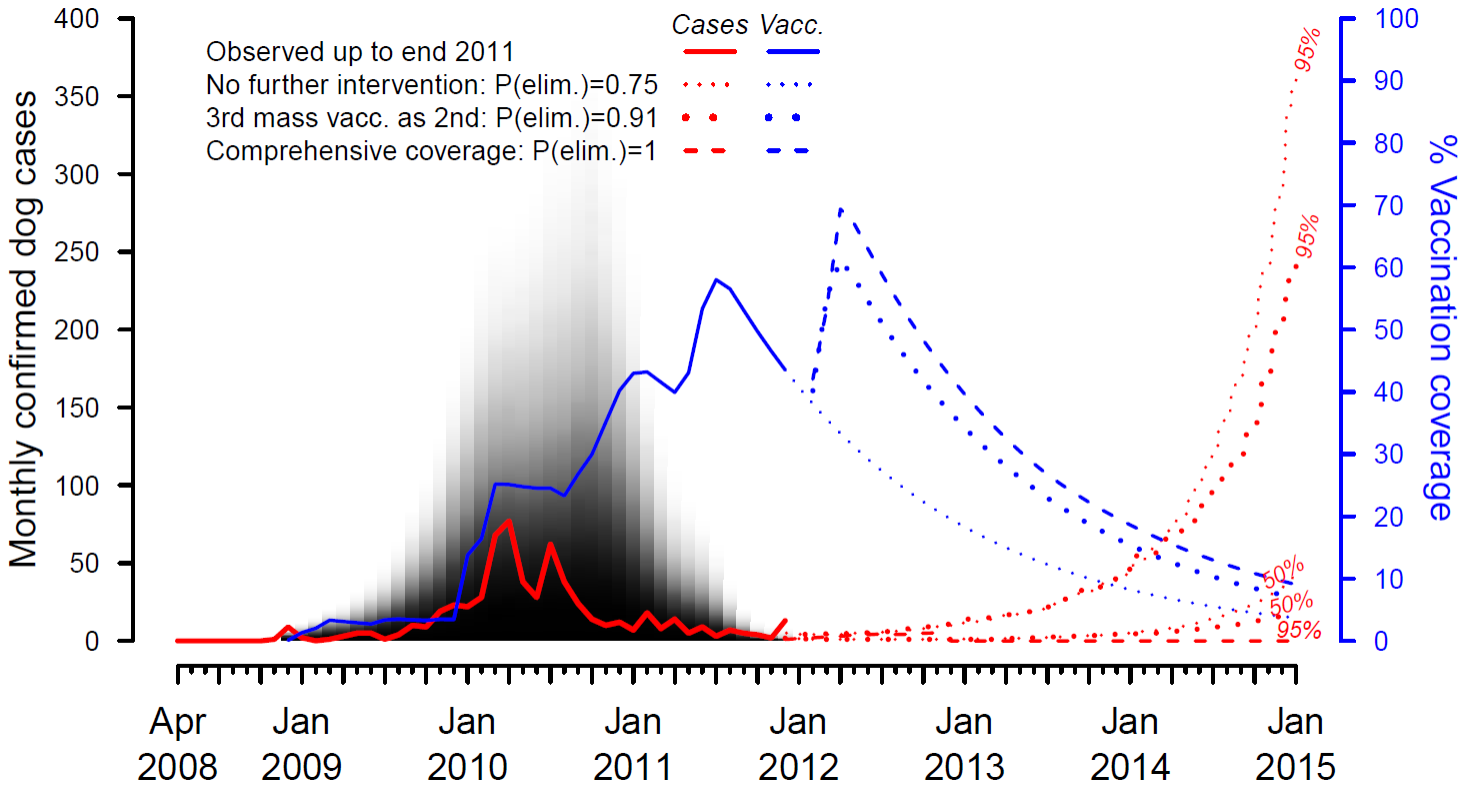
The vaccination campaigns on Bali and prospects for rabies eradication. Observed confirmed dog cases up to December 2011 (solid red line) overlay model confirmed cases (grey area, shaded according to confidence level) simulated from estimated vaccination coverage in the Bali dog population (solid blue line) and assuming 0.07 probability of confirming a case [6]. For 3 scenarios, vaccination coverage was projected forward to December 2014 (broken blue lines), and implemented in the model to project upper percentile limits for confirmed cases (broken red lines) and the probability of island-wide eradication (see legend and Fig. 3Aiv). The increase in cases in Dec 2011 may have been due to a substantial improvement in surveillance involving follow up of suspected animal bite cases by outbreak investigation teams.

Incorporation of all recorded vaccination efforts on Bali was necessary to generate simulated epidemics that matched the observed epidemic trajectory (Fig. 6). This included initial localized low coverage vaccinations using locally produced vaccines that required 3-month boosters which nevertheless played an important role in building up coverage and slowing the momentum of the epidemic (Fig. 6). Control was subsequently achieved through improving the scale, coverage and orchestration of vaccination, including switching to a longer lasting vaccine (Fig. 1A): in late 2010 and early 2011 the first island-wide campaign achieved target coverages of 70%, although because the campaign took several months to implement, the average island-wide coverage was around 40% (with ongoing turnover and waning immunity continually eroding coverage, Fig. 4). A second campaign was completed later in 2011 building up island-wide coverage to around 60% (Fig. 6). The overall trajectory towards eradication appears very promising especially if gaps are addressed in a third campaign currently underway (Fig. 5Aiv & 6). However, if control measures lapse, there is a more than 30% chance that within three years rabies will re-emerge to an endemic situation (Fig. 5Aiv & 6) with around 55 human deaths per year occurring on the basis of the relationship between confirmed cases and human deaths (Fig. 2Av). Over a ten-year time horizon, under the best-case scenario of rapid eradication from Bali as a result of a 3^rd^ comprehensive coverage vaccination campaign, approximately 550 human rabies deaths would therefore be averted in contrast to the endemic situation. Whereas if control measures are maintained, but not to the level required for eradication, low levels of rabies persistence would avert around 440 human rabies deaths but would require indefinite administration of expensive post-exposure prophylaxes (∼$1.5 million/year). These calculations assume awareness of rabies and the availability of PEP remain the same as over the course of the epidemic to date.

## Discussion

There are strong incentives for carrying out a mass dog vaccination programme to eradicate rabies from Bali. More than 100 human deaths have occurred since the start of the outbreak in 2008 [24]. Costs for the provision of post–exposure vaccine to bite victims in 2010 alone exceeded USD$2 million and would remain high in an endemic situation. If rabies was eradicated by mass dog vaccination, and assuming bite incidence returns to pre-outbreak levels (one tenth those in 2010), then precautionary use of post–exposure vaccine would also be ten–fold lower (∼100,000 USD per year). Following official declaration of freedom from rabies (2 years with no detected cases under effective surveillance [25]) these costs should reduce to zero. Our results suggest that eradicating rabies from Bali through mass dog vaccination is feasible; it would prevent hundreds of human rabies deaths, save millions of dollars, alleviate the trauma and panic that is currently widespread in local communities, and mitigate potential impacts on Bali's tourist industry. We investigated operational aspects of vaccination strategies to determine which are most critical to achieving eradication rapidly.

Our *R*_0_ estimate of 1.2 for rabies in Bali is remarkably similar to estimates for canine rabies elsewhere, which range from 1 to 2 [18], despite population densities varying by an order of magnitude. Even under a range of assumptions about the timing and extent of reactive control measures following confirmation of rabies on Bali, *R*_0_ remains between 1 and 2. Indeed, improvements in surveillance on Bali during the first year of the epidemic would likely lead to *R*_0_ being overestimated rather than underestimated.

The low *R*_0_ observed on Bali challenges assumptions that canine rabies transmission depends on population density [12], [17]. The relationship between *R*_0_ and density is in many ways parallel to the functional responses in predator prey interactions in population ecology. Borrowing existing concepts from population ecology helps to embed epidemiological phenomena in a different context, and may be helpful in understanding possible mechanisms underlying this relationship. The (much studied) mechanisms underlying Type 2 functional responses in predator prey interactions would be an obvious starting point suggested by the analogy. While further investigation is required to understand this phenomenon, our results suggest that moderate reductions in dog density are unlikely to have any beneficial effects on rabies control. Dog population management is often a common component of rabies control programmes, either exclusively or in combination with dog vaccination. Such programmes should be aware that the mass culling or sterilisation of dogs may not be an effective means of controlling rabies, and that as long as a high proportion of the dog population can be reached with vaccination, rabies should be brought under control.

The sensitivity of vaccination success to *R*_0_ (Fig. 3A) highlights the importance of estimating *R*_0_ locally and accurately and the need to prioritize surveillance including collection of incidence data. Overall, the low *R*_0_ suggests that only 17% of the population would need to be vaccinated to control rabies (*P_crit_* = 1−1/*R*_0_) [12], [17]. However, when realistic operational features are taken into account, particularly the pulsed nature of vaccination campaigns, and the birth of susceptible dogs, we find that coverage of less than 30% may never achieve eradication (Fig. 3B). At least 40% of dogs must be vaccinated to maintain island–wide coverage above 17% at all times (Fig. 4) and consecutive annual campaigns are needed to ensure eradication given the stochastic nature of rabies spread (Figs. 3 & 4). With annual comprehensive vaccinations achieving uniformly high coverage of at least 70% as recommended by WHO [12], [17] we would expect rabies to be eradicated from Bali within 1–3 years of initiating comprehensive vaccinations (Fig. 3B).

While we find that achieving high vaccination coverage is a decisive factor for disease elimination, follow–up campaigns are essential for achieving eradication, especially when achieving high coverage is problematic. At lower coverage, rapid population turnover and use of vaccines that confer only short–lived immunity could cause population–level protection to fall below *P_crit_* and reduce or preclude the chance of eradication (Fig. 4). Therefore use of long–acting vaccines particularly in populations with high turnover is recommended (Fig. 3C&D). We found a positive effect of six–month intervals between campaigns (Fig. 5Aii) probably because coverage levels were maintained above *P_crit_* for longer than with equivalent effort in back–to–back campaigns [26]. Our results highlight that a successful vaccination programme requires comprehensive and even coverage. Missing randomly distributed small pockets (totaling <10% of the total area) may not be overly detrimental, but omitting an equivalent contiguous area such as an administrative unit, could jeopardize an entire programme. Hence, mass vaccination programmes which are not perfectly implemented everywhere are of less concern than lack of participation from all communities.

High intensity mass vaccinations conducted over short periods that eradicated rabies from other regions [27] raised concerns about the need to complete campaigns on Bali as rapidly as possible. Our findings suggest taking longer to vaccinate a population (six–months versus one–month) has little impact on the success of otherwise equivalent campaigns, thus easing considerably the otherwise daunting logistical and financial challenges of synchronized mass vaccination campaigns [28]. In practice, increasing the speed with which a campaign is delivered might result in trade–offs if, for example, constraints include availability of personnel. Such logistical considerations are important: for instance, a slower six–month, but higher coverage (70%) campaign takes the same time to eradicate rabies as a one–month synchronized lower coverage (60%) campaign (Fig. 5C). Taking longer to reach more dogs will have a greater impact than achieving low coverage quickly, offering further optimism that eradication is still feasible where resources are limited or hard to synchronize (e.g. community–based).

In terms of spatial roll out, there may be advantages to starting vaccinations where an outbreak began, because this is probably where there are most cases and is the most intuitive starting point for policy makers. However, this may only improve the chances of success if long distance human-mediated dog movement is restricted. The reactive strategy emphasizes this point: with no long distance transport, eradication times were fastest using this strategy because the most infected areas were vaccinated repeatedly. Yet with frequent long distance transport (5%, and as was estimated on Bali) the reactive strategy performed worse than all others. Thus, while in some situations the reactive strategy could pay dividends, it is risky for at least two reasons: first, movement restrictions to slow rabies spread may be difficult to implement; and second, the potential to control an outbreak depends not only on the speed of transmission (*R*_0_ and dog movement), but also the quality of surveillance [6] and responsiveness of control measures [29]. In Bali surveillance was not in place before the incursion, which led to delays in initiating a response, and the culling of dogs caused some people to move their dogs to safer areas. Establishing national surveillance and emergency response procedures should be prioritized given the continuing spread of rabies in the region. Further investigation into the potential of reactive strategies is warranted, including contact tracing in focal areas of transmission, and modeling to predict undetected infections [30] and to identify locations posing the greatest risks [26]. Future data collection on the human transport of dogs would be valuable for modeling realistic patterns of spread that may help direct targeted vaccination.

Overall, our analyses strongly support the feasibility of rabies eradication from Bali and our modeling conclusions are borne out by the vaccinations campaigns carried out to date (Fig. 6). Whilst logistical difficulties of mobilization and implementation proved challenging, and heterogeneities in coverage compromised overall effectiveness, the extensive vaccination campaigns conducted have brought the epidemic under control. Further campaigns will be needed to eradicate rabies from Bali, and improving the comprehensiveness of these campaigns should be a high priority to achieve this goal. Once rabies does reach very low levels, then control measures may lapse and the risk of new incursions becomes an obvious danger, which we have not considered here. These risks are being evaluated in on-going field and modelling studies but, in the long term, genetic data could provide valuable information about the frequency and source of incursions. Eradication of rabies from Bali would not only save hundreds of lives, and millions of dollars by mitigating the indefinite need for expensive post-exposure prophylaxis, but would provide a valuable precedent for the feasibility of rabies eradication in very large and dense dog populations through effectively conducted mass vaccinations.

More generally we make the following practical recommendations: 1) There is no evidence that rabies transmission in domestic dogs is density dependent over commonly encountered ranges of dog densities, so controlling rabies in higher density dog populations should not require higher vaccination coverage; 2) Vaccines that provide at least one year of protection should be effective, but the use of vaccines of shorter duration that require a booster could compromise the effectiveness of vaccination campaigns; 3) The advantages to spatially strategic roll-out or intensified synchronous effort for implementing vaccination campaigns are not justified if the increased logistical challenges compromise coverage; 4) Human–mediated transport of dogs expedites the spread of rabies and vaccination performance could be improved by restricting dog movement. However, there is currently no infrastructure to achieve this on Bali and indeed some dog owners in Bali reportedly moved animals to avoid culling or to replace dogs that had been culled, which could jeopardize spatial targeting of vaccination; 5) While achieving high coverage ensures the best possible chance of rabies eradication, repeat campaigns are vital to guarantee this. 6) The greatest concern for eradication programmes would be the lack of participation from any administrative areas, for example in Bali, omission of even the smallest of the nine Regencies that consists of 59 villages or 6% of the island could dramatically reduce the odds of achieving eradication to one third or less (Fig. 5D). Our findings about the impact of omitting contiguous subpopulations may help explain why eradicating disease is so difficult without comprehensive coverage, particularly in landlocked areas with recurrent introductions from neighboring populations [3], [4], [26]. Determining the impact of neighbouring endemic areas on the effort required to eradicate rabies is an important question to address in future studies. Nonetheless, our results further emphasize the need for regional coordination in large–scale control programmes, as evidenced by successful control of rabies in the Americas [31] in contrast to Africa [16].

## Supporting Information

Text S1 Additional details on the model and simulation videos.
(DOCX)

Video S1 Simulation of a rabies outbreak on Bali and vaccination using the ‘rotate’ strategy.
(AVI)

Video S2 Simulation of a rabies outbreak on Bali and vaccination using the ‘react without repeat’ strategy.
(AVI)

Video S3 Simulation of a rabies outbreak on Bali and vaccination using the ‘random’ strategy with 544 cells (selected randomly) left unvaccinated, which is a roughly equivalent area to 2 blocks (∼8% of the island).
(AVI)

Video S4 Simulation of a rabies outbreak on Bali and vaccination using the ‘random’ strategy with 2 blocks left unvaccinated (∼8% of the island).
(AVI)

Video S5 Simulation of a rabies outbreak on Bali and vaccination as implemented in Bali up to the end of 2011. The simulation was allowed to continue without further vaccination which, in this case, results in re-emergence of rabies as vaccination coverage wanes.
(AVI)

Video S6 Simulation of a rabies outbreak on Bali and vaccination in the model as implemented in Bali up to the end of 2011, followed by a comprehensive 70% coverage 3^rd^ vaccination round in 2012. This simulation results in elimination of rabies from Bali.
(AVI)
