# Supplementary material for: Designing Programs for Eliminating Canine Rabies from Islands: Bali, Indonesia as a Case Study
Source: PLoS Negl Trop Dis. 2013 Aug 22;7(8):e2372. doi: 10.1371/journal.pntd.0002372 (PMC3749988; doi:10.1371/journal.pntd.0002372)
Supplement: Text S1 — Additional details on the model and simulation videos. (DOCX) [file pntd.0002372.s001.docx]

#### Supplementary Information

To accompany: ‘Designing programs for eliminating canine rabies from islands: Bali, Indonesia as a case study’ by Townsend et al.

**Further details of the model**

- New infectious cases could only be allocated a terrestrial grid cell.
- Percentile interval: Confidence intervals are based on assuming a variable is distributed according to a standard distribution such as Gaussian or binomial, whereas percentile intervals are based on an ordered list of the observed values of the variable. A 95 percentile interval is given by the 5^th^ and 95^th^ percentile of the variable, which are the values below which 5% and 95% of the observations may be found.
- We did not model an additive effect of vaccination coverage from previous campaigns i.e. if 70% of the population was vaccinated previously, but coverage dropped to 40% during the inter-campaign interval, a subsequent campaign reaching 70% of dogs only boosts coverage back to 70%. This is equivalent to assuming that previously vaccinated dogs are revaccinated in addition to vaccinating new dogs to bring coverage up to 70%.
- We assume no further introductions of rabies into Bali after the initial incursion.

**Videos S1-6** are example model simulations of the rabies epidemic on Bali and the impact of a range of the dog vaccination strategies explored in the paper. The red dot marks the incursion location and yellow dots indicate cells that were infected in the last month. Vaccination is represented by cell color, with more opaque white corresponding to higher coverage. The simulation was generated using parameters in Table 1 and mass vaccination strategies as described in Table 2. In Videos S5-S6 lakes, reservoirs, forested areas and mountain peaks were assumed to be uninhabited by dogs and are block colored white in the simulations.
